# Hepatitis A Outbreak Characteristics: A Comparison of Regions with Different Vaccination Strategies, Spain 2010–2018

**DOI:** 10.3390/vaccines9111214

**Published:** 2021-10-20

**Authors:** Angela Domínguez, Carmen Varela, Núria Soldevila, Conchita Izquierdo, María Guerrero, Marina Peñuelas, Ana Martínez, Pere Godoy, Eva Borràs, Cristina Rius, Núria Torner, Ana María Avellón, Jesús Castilla, PREVICET Working Group on Viral Hepatitis

**Affiliations:** 1 Department of Medicine, Universidad de Barcelona, 08036 Barcelona, Spain; angela.dominguez@ub.edu; 2 CIBER Epidemiología y Salud Pública (CIBERESP), Instituto de Salud Carlos III, 28029 Madrid, Spain; mvarelam@isciii.es (C.V.); a.martinez@gencat.cat (A.M.); pere.godoy@gencat.cat (P.G.); eva.borras@gencat.cat (E.B.); crius@aspb.cat (C.R.); nuriatorner@ub.edu (N.T.); aavellon@isciii.es (A.M.A.); jcastilc@navarra.es (J.C.); 3 Centro Nacional de Epidemiología, Instituto de Salud Carlos III, 28029 Madrid, Spain; m.guerrero@isciii.es (M.G.); marina.penuelas@isciii.es (M.P.); 4 Agència de Salut Pública de Catalunya, 08005 Barcelona, Spain; conchita.izquierdo@gencat.cat; 5 Agència de Salut Pública de Barcelona, 08023 Barcelona, Spain; 6 Centro Nacional de Microbiología, Instituto de Salud Carlos III, 28222 Madrid, Spain; 7 Instituto Salud Pública de Navarra-IdiSNA, 31003 Pamplona, Spain

**Keywords:** Hepatitis A, outbreak, transmission, vaccination strategy, risk groups

## Abstract

We compared the cumulative incidence and characteristics of hepatitis A outbreaks in two groups of Spanish autonomous regions according to whether a universal or risk group vaccination strategy was followed. Outbreaks between 2010 and 2018 were analyzed. The cumulative incidence rate of outbreaks was estimated and compared by estimating the rate ratio (RR). The characteristics of the outbreaks and those of the first cases were compared. Adjusted OR (aOR) were calculated using a multivariate logistic regression model. Outbreak incidence was 16.04 per million persons in regions with universal vaccination and 20.76 in those with risk-group vaccination (RR 0.77; 95%CI 0.62–0.94). Imported outbreaks accounted for 65% in regions with universal vaccination and 28.7% in regions with risk-group vaccination (aOR 3.88; 95%CI 2.13–7.09). Adolescents and young adults aged 15–44 years and men who have sex with men were less frequently the first case of the outbreak in regions with a universal vaccination strategy (aOR 0.54; 95%CI 0.32–0.92 and 0.23; 95%CI 0.07–0.82, respectively). The cumulative incidence rate of outbreaks was lower in regions with universal vaccination. In all regions, independently of the vaccination strategy, activities to vaccinate persons belonging to high-risk groups for infection should be emphasized.

## 1. Introduction

Hepatitis A, an acute disease caused by the hepatitis A virus, an RNA virus of the *Hepatovirus* genus and *Picornaviridae* family [1], causes tens of millions of annual infections worldwide. In 2017, it was estimated there were 170 million new cases [2], and the World Health Organization (WHO) estimates that the 7134 hepatitis A deaths in 2016 accounted for 0.5% of all deaths due to viral hepatitis [3]. The lowest-income developed countries have a high incidence rate but a low disease burden. The most important determinant of the likelihood of clinical expression is the age at which infection occurs: most infections in children aged <5 years are silent, and the proportion of symptomatic infections increases in adolescents and adults [4].

The highest-income countries have a very low incidence rate, but persons who become infected have a high likelihood of hospitalization and risk death from acute liver failure [5]. Although unusual in low endemic countries, hepatic failure requiring transplantation and death from hepatitis A may also occur [6].

In Spain and most countries of western Europe, the disease incidence is generally low, but outbreaks occur each year. In a study of 46 hepatitis A adult patients in the context of an outbreak in Barcelona, 18 patients (39%) required hospitalization, 9 (20%) presented severe acute hepatitis (none presented hepatic encephalopathy), and the remaining 19 had gastrointestinal symptoms or dehydration; one patient was admitted to the intensive care unit due to thrombotic microangiopathy [7].

The WHO recommends that hepatitis A vaccination should be included in the national immunization schedule in children aged ≥1 year, according to the country’s incidence of acute hepatitis A, a change in the endemicity from high to intermediate, and consideration of cost-effectiveness. The use of hepatitis A vaccination rather than passive prophylaxis with immune globulin is recommended for pre-exposure prophylaxis (e.g., for travelers to areas of higher hepatitis A endemicity) and post-exposure prophylaxis (e.g., for close contacts of acute cases of hepatitis A) [8]. In Spain, hepatitis A vaccination is recommended for risk groups only, and hepatitis A vaccination as post-exposure prophylaxis is recommended for people aged <50 years [9]. However, some Spanish regions or autonomous cities, such as Catalonia and Ceuta, after considering the epidemiological situation and the potential benefits of universal vaccination, have opted for the universal hepatitis A vaccination strategy in addition to the vaccination of risk groups [10,11]. Catalonia adopted this strategy in 1999 for pre-adolescents and at 12 months and 6 years in children in 2014; Ceuta has applied the strategy since 2000, with vaccination at 15 and 24 months and 13 years. In all hepatitis A cases, vaccination is offered free of charge.

The objective of this study was to compare the cumulative incidence and characteristics of hepatitis A outbreaks during 2010–2018 in two groups of Spanish autonomous regions or cities according to the universal or risk-group hepatitis A vaccination strategy followed.

## 2. Methods

### 2.1. Outbreak and Case Reporting

In Spain, hepatitis A cases and outbreaks of any etiology must be statutorily reported to the regional and national public health authorities [12]. Laboratory-confirmed and probable (a clinically-compatible case epidemiologically linked to another confirmed case or source of infection) cases of hepatitis A must be reported to the National Epidemiological Surveillance Network. An outbreak of hepatitis A was defined as ≥2 epidemiologically linked hepatitis A cases. All hepatitis A outbreaks from Castile and Leon, Catalonia, Ceuta, Community of Valencia, Madrid, Murcia, and Navarre between 2010 and 2018 reported to the National Center of Epidemiology were included. The population of these regions in January 2015 was 23,376,360 (50.32% of the Spanish population). An imported case was considered as a hepatitis A case with a history of travel outside Spain 15–50 days prior to symptom onset.

### 2.2. Variables

The following data were collected for each outbreak: year of occurrence; mode of transmission (common source or person-to-person), setting; whether the outbreak was caused by an imported case (yes or no); number of exposed, affected, and hospitalized people (median and range); immunoglobulin or vaccine recommendation to exposed people (yes or no); and the number of exposed people who received immunoglobulin or vaccine (median and range). The following characteristics of the first case of the outbreak were collected: age (0–14, 15–44, and ≥45 years), sex, born in Spain or not, occupation, history of hepatitis A vaccination (yes or no), recent history of travel to another country (yes or no), being a man ≥15 years who has sex with men (MSM) (yes or no), and being a drug user (in aged ≥15 years).

The general characteristics of outbreaks and characteristics of the first case of the outbreak corresponding to outbreaks in regions or autonomous cities with a universal vaccination strategy (Catalonia and Ceuta, with a population of 7,481,683) and five regions with a risk-group vaccination strategy (Castile and Leon, Community of Valencia, Madrid, Murcia, and Navarre, with a population of 15,894,677) were compared.

### 2.3. Statistical Analysis

Cumulative incidence rates of outbreaks per 1,000,000 persons were calculated for the two study groups and compared using the rate ratio (RR) with 95% confidence intervals (CI). The annual incidence rate of reported cases per 100,000 persons was also estimated.

The characteristics of the outbreaks and of the first cases in the two study groups were compared using the chi-square test for categorical variables and the Mann–Whitney U test for continuous variables.

The crude and adjusted odds ratios (OR) and their 95% confidence intervals were estimated using logistic regression. Adjusted OR (aOR) were calculated using a multivariate logistic regression model after adjusting for significant variables in the bivariate analysis in the characteristics of outbreaks model and for the variables age and sex in the characteristics of the first case model.

The analysis was made using IBM SPSS Statistics for Windows v.25 (IBM Corporation, Armonk, NY, USA).

## 3. Results

We included 450 outbreaks: 120 in regions with a universal vaccination strategy and 330 in regions with a risk-group vaccination strategy. The cumulative incidence rate of outbreaks was 19.25 per million persons in all regions studied, 16.04 in regions with universal vaccination, and 20.75 in regions with risk-group vaccination (RR 0.77; 95%CI 0.62–0.95; *p* = 0.01). The cumulative incidence rate per million of person-to-person outbreaks and of outbreaks in the family/household setting was lower in regions with universal vaccination (RR 0.67; 95%CI 0.53–0.85; *p* = 0.001 and RR 0.74; 95%CI 0.58–0.94; *p* = 0.01, respectively). Conversely, the cumulative incidence of imported outbreaks was higher in regions with universal vaccination (RR 1.62; 95%CI 1.13–2.33; *p* = 0.01) (Table 1).

The annual incidence rate of reported cases in all the regions studied ranged between 1.36 per 100,000 in 2012 to 9.22 in 2017. In regions with a universal vaccination strategy, the incidence rate was lowest in 2015 (1.06 per 100,000) and highest in 2017 (5.26 per 100,000). In regions with a risk-group vaccination strategy, rates ranged between 1.32 per 100,000 in 2012 to 11.12 in 2017.

**Figure 1:**
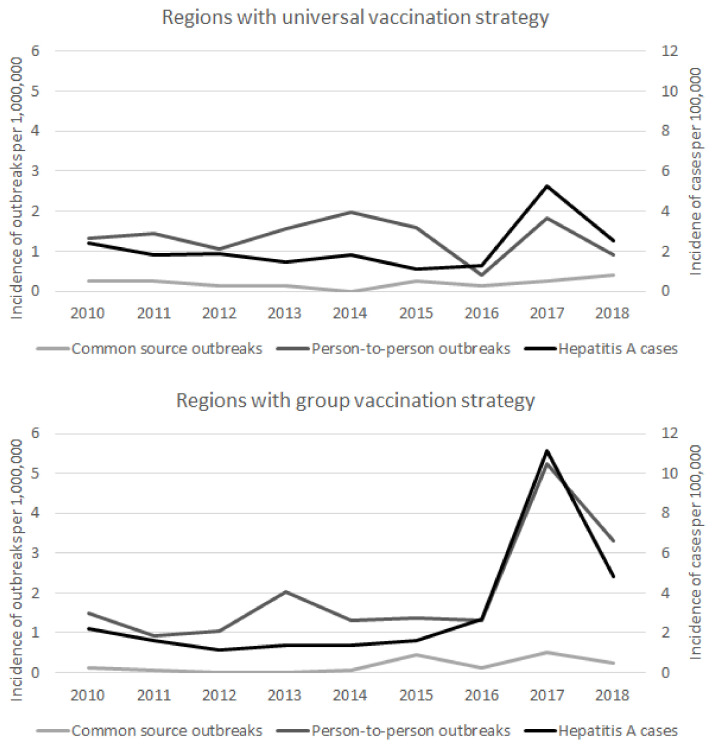
Annual incidence rate of hepatitis A cases and hepatitis A outbreaks according to transmission mode by vaccination strategy. Spain, 2010–2018.

There was an increase in the cumulative incidence of person-to-person outbreaks and reported cases in 2017 and 2018, mainly in regions with risk-group vaccination but also in regions with universal vaccination. The yearly evolution of outbreaks and reported cases in the study regions is shown in Figure 1.

Common source outbreaks were non-significantly more frequent in regions with universal vaccination (13.2%) than in those with risk-group vaccination (7.9%) (Table 2). Sixty-five percent of outbreaks were classified as imported in regions with a universal vaccination strategy compared with 28.7% in those with a risk-group strategy (aOR 3.88; 95%CI 2.13–7.09; *p* < 0.001).

The median of hospitalized patients was lower in regions with universal vaccination (0; range 0–5) than in those with risk-group vaccination (1, range 0–47). Considering only common-source outbreaks (Table 3), no significant differences were observed between the two study groups. In person-to-person outbreaks, imported outbreaks were more frequent in regions with universal vaccination (aOR 4.38; 95%CI 2.29–8.35, *p* < 0.001), and the median of hospitalized patients was lower (*p* = 0.03) in regions with universal vaccination (0; range 0–3) than in those with risk-group vaccination (1, range 0–47). No deaths were recorded in any outbreak. No significant differences were observed as to whether immunoglobulin or vaccine was recommended for post-exposure prophylaxis.

With respect to the characteristics of the first case of the outbreak, in all types of outbreaks (Table 4), persons aged 15–44 years were less frequent in regions with universal vaccination strategy than in those with risk-group vaccination (aOR 0.54; 95%CI 0.32–0.92; *p* = 0.02). The first case was less frequently an MSM in regions with universal vaccination than in regions with risk-group vaccination both in all outbreaks and person-to-person outbreaks (aOR 0.23; 95%CI 0.07–0.82; *p* = 0.02 and aOR 0.13; 95%CI 0.03–0.55; *p* = 0.006, respectively). No significant differences were observed in the characteristics of the first case in common-source outbreaks (Table 5).

Due to the large increase observed in the incidence rate and person-to-person outbreaks in 2017 and 2018 because of MSM transmission, a specific analysis of data for 2010–2016 is shown in the supplementary tables. During 2010–2016, no differences were observed in the cumulative incidence of outbreaks in the two study groups. The cumulative incidence (Supplementary Table S1) of imported outbreaks was higher in regions with universal vaccination (RR 2.21; 95CI 1.48–3.31; *p* = 0.001). In person-to-person outbreaks, imported outbreaks were more frequent in regions with universal vaccination (aOR 3.42; 95%CI 1.68–6.94; *p* = 0.001) (Supplementary Table S2).

## 4. Discussion

The results of this nine-year study show that the cumulative incidence of hepatitis A outbreaks was lower in regions with universal vaccination than in those with high-risk vaccination. Even though asymptomatic infections are not recorded, and the incidence rate cannot be estimated accurately, epidemiological data on hepatitis A outbreaks can offer useful insights on the evolution of the disease epidemiology and may be used to prioritize future public health strategies [13].

A higher proportion of imported outbreaks was observed in regions with a universal vaccination strategy, which can be explained because universal vaccination protects the indigenous population. Immigrants making a recent trip to visit friends and relatives is a frequent risk factor for an outbreak, and various authors have reported the importance of vaccinating people traveling to endemic countries [14,15,16,17].

We recorded a lower median number of hospitalizations in regions with a universal vaccination strategy in person-to-person outbreaks but not in common-source outbreaks.

The fact that the proportion of cases 15–44 years is higher in regions with high-risk vaccination than in regions with universal vaccination might explain why there were more severe clinical presentations and more hospitalizations. Other authors have reported that patients aged >30 years have higher complication rates of renal failure or prolonged cholestasis than those aged <30 years [17].

Hospitalization is an important health consequence due to the consumption of health resources and the accompanying economic costs [18,19]. Universal hepatitis A vaccination was introduced in 2008 in Greece [20], and hepatitis A hospitalization rates decreased significantly between the pre-vaccination (1999–2008) and post-vaccination (2009–2013) eras, falling from 50.5 to 20.8/1000 hospitalizations [20].

In a 2015 hepatitis A outbreak associated with an Irish childcare facility that involved 12 symptomatic cases, of whom six adult cases required hospitalization, had associated costs of control were estimated at €43,400–€47,400. Plunkett et al. [21] carried out a study in England to estimate the burden associated with an outbreak of hepatitis A affecting mainly (but not exclusively) MSM that involved 670 confirmed cases, and the associated healthcare costs were estimated to be approximately £1,500,000.This illustrates the considerable adult morbidity that may be involved hepatitis A outbreaks and highlights the challenges in controlling the morbidity associated with an outbreak, early interventions to administer post-exposure prophylaxis, and the importance of early recognition of hepatitis A by physicians [22].

Although no death was recorded in our study, hepatitis A-related mortality has been described in patients with pre-existing liver disease, diabetes, cardiovascular disease, or platelet disorders [23].

We found that people aged 15–44 years were less frequently the first case of the outbreak in regions with a universal vaccination strategy. This is concordant with the results obtained in the study by Torner et al. [24] and may be due to the fact that this age group benefited directly from universal vaccination programs in preadolescents in Ceuta since 2000 and in Catalonia from 1999 until 2014.

Studies have reported a major outbreak of hepatitis A that swept through European and American countries between mid-2016 and 2017, mainly within the MSM community [25,26,27,28]. Bauer et al. [6], in a study carried out in Vienna coinciding with a multinational hepatitis A outbreak in MSM, reported 53.6% of patients were hospitalized with severe disease, 9% required ICU support, and one patient received a liver transplant within 30 days. Spanish studies have found that recent hepatitis A outbreaks were closely related to a younger age and sexual practices [29,30]. Fortea et al. [31] studied the characteristics of hepatitis A patients in Cantabria (Spain) in 2013–2016 and 2017–2018 and found that there was a greater proportion of MSM during the second period. In the present study, when these two years are excluded from the analysis, no differences were observed in the cumulative incidence of outbreaks in the two study groups.

We found that MSM were less frequently the first case of an outbreak in regions with universal vaccination. Despite international and national public health recommendations, [8,9,32] adherence to these recommendations remain difficult to attain. Tortajada et al. [33] reported that, in spite of a specific program targeting gay bathhouses, the disease incidence was not modified, and new outbreaks were not prevented. Some authors [34] have emphasized that proactive rather than reactive control strategies, including continuous vaccination of high-risk groups, particularly in urban centers, which can serve as hubs for disease transmission, may be effective in controlling future outbreaks that may spill over into the general population and that healthcare professionals should work towards vaccination of populations with risk factors.

As stated, hepatitis A outbreaks may also occur in regions with universal vaccination. In the USA, despite a significant decrease in hepatitis A incidence rates after 2006 when the hepatitis A vaccine was recommended for all children aged 12–23 months and for persons at increased risk of infection, person-to-person outbreaks continue to occur among the population at risk for hepatitis A who are unvaccinated [32]. A large outbreak affecting MSM in San Diego (USA) involved 590 confirmed cases between 2016 and 2018, with 69% hospitalization and 3.4% fatality rate. A state of emergency was declared, and the public health strategy included more than 160,000 vaccinations, health measures, and education with innovative and highly individualized strategies in an attempt to ensure vaccination of those at highest risk. Vaccination was the most important strategy in stopping the outbreak, but the problem of vulnerable populations and other social determinants of health should probably also be addressed [35]. In Apulia (Italy), where an active free immunization program against hepatitis A targeted at infants aged 15–18 months and adolescents started in 1998, the effects of routine immunization were evident in the targeted cohorts but not in people from other cohorts with high rates of susceptibility [36].

Gozlan et al. [37] found that in Israel, despite a vaccination program introduced in 1999 with two doses at 1.5 and 2 years, hepatitis A could still be transmitted to unvaccinated, high-risk populations, such as MSM, and called for the introduction of strategies aimed at increasing vaccination coverages in specific risk groups. Yoon et al. [17] proposed reinforcing hepatitis A vaccination with nationwide campaigns to advance towards disease eradication. Hepatitis A has been considered as a candidate disease for eradication because it fulfils most of the required characteristics, and very effective vaccines are available, but international bodies have not made this recommendation due to cost and feasibility [38].

Most recent studies assessing the efficiency of routine vaccination strategies in low or very low endemicity countries fail to demonstrate a benefit with routine hepatitis A vaccination. Ghildayal et al. [19] carried out economic analyses of routine vaccination in the USA and concluded that because the cost of routine vaccination was $55,778 per QALY saved compared with no vaccination, and this cost was under the accepted threshold of $100,000, universal vaccination should be considered cost-effective. In Switzerland, Fahrni et al. [15] found that the most cost-effective vaccination strategy was an individualized approach based on the age and origin of individuals, reducing costs by 17% compared with systematic vaccination. In a systematic review by Wong et al. [39] of studies carried out in Hong-Kong on cost-effectiveness of vaccine-preventable diseases, it was concluded that hepatitis A routine vaccination was not cost-saving. Studies of the efficiency of other immunization strategies also failed to demonstrate an economic benefit [40,41]. A cost-utility analysis carried out in Ontario (Canada) comparing expanded high-risk, publicly funded hepatitis A vaccination providing free vaccine for travelers to endemic regions concluded that this expanded program substantially exceeded the accepted cost-effectiveness threshold of 50,000 Canadian dollars per QALY gained [41]. In the USA, a catch-up hepatitis A vaccination would be less cost-effective than many other vaccine interventions. The cost per QALY gained would range from $269,000–$725,000, varying with age at catch-up vaccination and becoming cost effective at a threshold of $50,000 per QALY only when incidence of hepatitis A rose to about 5.0 cases per 100,000 population [40].

Unfortunately, there are no recent published studies carried out in Spain about the cost-effectiveness of different strategies for hepatitis A vaccination [42] and it is necessary to conduct a cost-effectiveness study to assess the potential benefit of including the vaccine into the routine immunization calendar.

Coordinating hepatitis A vaccination campaigns with other services, such as public communication campaigns through social media and social services, may further improve access to hepatitis A vaccination for high-risk individuals [43,44]. The detailed study of activities to detect and investigate hepatitis A outbreaks may be useful to generate evidence-based operational lessons for the future [45,46]. Sharp et al. [44] compared two community outbreaks by returning travelers in England, which were managed by independent control teams. In one outbreak, mass vaccination was deployed in the residential area and two schools, while in the other, vaccination was reserved for household-type contacts of cases. The authors state that mass vaccination in the first outbreak may have shortened its length and offered a public benefit to the community, but no conclusion could be drawn about the overall effectiveness of mass vaccination to control outbreaks of hepatitis A in low-endemicity settings. Hepatitis A vaccination is also recommended as post-exposure prophylaxis in people under 40, 50, or 60 years of age in different countries or regions [47], and we found no differences in the level of recommendation of vaccination as post-exposure prophylaxis in regions with universal or risk-group vaccination strategies.

The study has some limitations. Firstly, the retrospective nature impairs the capacity to obtain some relevant data, such as the age distribution of the persons affected in each outbreak. Second, information on the duration of hospitalizations is lacking, and this could be useful in assessing the burden of outbreaks. Third, due to the retrospective design, the amount of information was not available for several variables, such as whether the outbreak was imported, whether post-exposure measures were recommended, and the number of exposed people to whom vaccine or immunoglobulin was administered.

## 5. Conclusions

This study highlights the burden of hepatitis A outbreaks in Spain. The cumulative incidence rate of outbreaks was lower in regions with a universal vaccination strategy than in regions with a risk-group vaccination strategy. MSM were less frequently the first case of the outbreak in regions with a universal vaccination strategy; imported outbreaks were more frequent in regions with universal vaccination. Cost-effectiveness studies of different hepatitis A immunization strategies in Spain are needed.

Regardless of the vaccination strategy, activities aimed at proactively vaccinating persons in groups at high risk of infection, such as MSM and travelers to endemic countries, including immigrants and their children, should be improved.

## Figures and Tables

**Table 1:** Number and cumulative incidence rate per million inhabitants of hepatitis A in regions with different vaccination strategies. Spain, 2010–2018.

	Regions with Universal Vaccination Strategy *n* = 120 *n* (CIR)	Regions with Risk-Group Vaccination Strategy *n* = 330 *n* (CIR)	Rate Ratio (95% CI)	*p*-Value
Outbreak incidence	16.04	20.75	0.77 (0.62–0.95)	0.01
Transmission mode				
Common source	14 (1.87)	25 (1.57)	1.19 (0.60–2.28)	0.60
Person-to-person	92 (12.30)	290 (18.24)	0.67 (0.53–0.85)	0.001
Setting				
Family/household	93 (12.43)	266 (16.74)	0.74 (0.58–0.94)	0.01
School	7 (0.94)	23 (1.45)	0.65 (0.26–1.46)	0.31
Restaurant/facility canteen	0 (0)	8 (0.50)	0.12 (0.01–2.16)	0.19
Leisure facility/summer camp	1 (0.13)	2 (0.13)	1.06 (0.04–13.97)	0.96
Community/other/various	6 (0.80)	26 (1.64)	0.49 (0.18–1.14)	0.10
Other closed facilities	0 (0)	3 (0.19)	0.30 (0.02–5.87)	0.63
Imported outbreak	52 (6.95)	68 (4.28)	1.62 (1.13–2.33)	0.01

CIR, cumulative incidence rate.

**Table 2:** Characteristics of hepatitis A outbreaks in regions with different vaccination strategies. Spain, 2010–2018.

	Regions with Universal Vaccination Strategy *n* = 120	Regions with Risk-Group Vaccination Strategy *n* = 330	Crude Odds Ratio (95% CI)	*p*-Value	Adjusted Odds Ratio (95% CI)	*p*-Value
Transmission mode						
Common source	14 (13.2%)	25 (7.9%)	1.76 (0.88–3.54)	0.11	1.54 (0.64–3.68)	0.34
Person-to-person	92 (86.8%)	290 (92.1%)	Ref.		Ref.	
Setting						
Family/household	93 (86.9%)	266 (81.1%)	Ref.		Ref.	
School	7 (6.5%)	23 (7.0%)	0.87 (0.36–2.10)	0.76	1.67 (0.54–5.18)	0.37
Restaurant/facility canteen	0 (0%)	8 (2.4%)	-		-	
Leisure facility/summer camp	1 (0.9%)	2 (0.6%)	1.43 (0.13–15.96)	0.77	2.03 (0.15–27.44)	0.59
Community/other/various	6 (5.6%)	26 (7.9%)	0.66 (0.26–1.65)	0.37	0.73 (0.23–2.31)	0.59
Other closed facilities	0 (0%)	3 (0.9%)	-		-	
Imported outbreak						
Yes	52 (65.0%)	68 (28.7%)	4.62 (2.69–7.91)	<0.001	3.88 (2.13–7.09)	<0.001
No	28 (35.0%)	169 (71.3%)	Ref.			
No. of exposed people *, median (range)	5 (2–422)	5 (2–35240)		0.24		0.77
No. of affected people, median (range)	2 (2–218)	2 (2–88)		0.52		0.22
No. of hospitalized cases ^†^, median (range)	0 (0–5)	1 (0–47)		<0.001		0.07
IG recommended to exposed people						
Yes	21 (31.3%)	16 (32.7%)	0.94 (0.43–2.07)	0.88	2.16 (0.67–6.97)	0.20
No	46 (68.7%)	33 (67.3%)	Ref.		Ref.	
Vaccine recommended to exposed people						
Yes	81 (92.0%)	230 (95.0%)	0.60 (0.23–1.59)	0.31	0.44 (0.11–1.71)	0.23
No	7 (8.0%)	12 (5.0%)	Ref.		Ref.	
No. of exposed people administered IG ^‡^, median (range)	0 (0–68)	0 (0–80)		0.79		0.76
No. of exposed people administered vaccine ^§^, median (range)	3 (0–482)	3 (0–672)		0.36		0.11

IG, immunoglobulin; Information was not available in * 87, ^†^ 27, ^‡^ 285, ^§^ 222 outbreaks.

**Table 3:** Characteristics of common source and person-to-person hepatitis A outbreaks in regions with different vaccination strategies. Spain, 2010–2018.

	Regions with Universal Vaccination Strategy *n* = 120	Regions with Risk-Group Vaccination Strategy *n* = 330	Crude Odds Ratio (95% CI)	*p-*Value	Adjusted Odds Ratio (95% CI)	*p-*Value
COMMON SOURCE OUTBREAKS						
Setting						
Family/household	12 (92.3%)	20 (87.0%)	-		-	
Restaurant/facility canteen	0 (0%)	3 (13.0%)	-		-	
Community/other/various	1 (7.7%)	0 (0%)	-		-	
Imported outbreak	7 (53.8%)	10 (47.6%)	1.28 (0.32–5.13)	0.72	1.67 (0.33–8.47)	0.53
No. of exposed people, median (range)	5 (2–38)	5 (2–400)		0.92		0.82
No. of affected people, median (range)	2 (2–38)	2 (2–31)		0.59		0.49
No. of hospitalized cases, median (range)	0 (0–5)	1 (0–16)		0.38		0.65
PERSON-TO-PERSON TRANSMISSION OUTBREAKS						
Setting						
Family/household	71 (86.6%)	233 (80.3%)	Ref.		Ref.	
School	6 (7.3%)	22 (7.6%)	0.90 (0.35–2.29)	0.82	1.69 (0.65–6.59)	0.22
Restaurant/facility canteen	0 (0%)	5 (1.7%)	-		-	
Leisure facility/summer camp	1 (1.2%)	2 (0.7%)	1.64 (0.15–18.36)	0.69	2.39 (0.18–32.34)	0.51
Community/other/various	4 (4.9%)	25 (8.6%)	0.53 (0.18–1.56)	0.25	0.44 (0.10–2.04)	0.29
Other closed facilities	0 (0%)	3 (1.0%)	-		-	
Imported outbreak	36 (62.1%)	54 (26.2%)	4.61 (2.49–8.52)	<0.001	4.38 (2.29–8.35)	<0.001
No. of exposed people, median (range)	5 (2–422)	5 (2–473)		0.44		0.46
No. of affected people, median (range)	2 (2–218)	2 (2–88)		0.58		0.71
No. of hospitalized people, median (range)	0 (0–3)	1 (0–47)		<0.001		0.03

**Table 4:** Characteristics of the first case in hepatitis A outbreaks in regions with different vaccination strategies. Spain, 2010–2018.

	Regions with Universal Vaccination Strategy *n* = 120	Regions with Risk-Group Vaccination Strategy *n* = 330	Crude Odds Ratio (95% CI)	*p*-Value	Adjusted Odds Ratio (95% CI)	*p-*Value
Age						
0–14 years	68 (58.6%)	86 (47.5%)	Ref.		Ref.	
15–44 years	36 (31.0%)	78 (43.1%)	0.58 (0.35–0.97)	0.04	0.54 (0.32–0.92)	0.02
≥45 years	12 (10.3%)	17 (9.4%)	0.89 (0.40–2.00)	0.78	0.83 (0.36–1.89)	0.65
Sex						
Male	77 (67.5%)	138 (72.3%)	Ref.		Ref.	
Female	37 (32.5%)	53 (27.7%)	1.25 (0.76–2.07)	0.38	1.13 (0.66–1.95)	0.65
Born in Spain						
Yes	36 (65.5%)	51 (67.1%)	0.93 (0.45–1.93)	0.84	1.10 (0.48–2.54)	0.81
No	19 (34.5%)	25 (32.9%)	Ref.		Ref.	
Occupation						
Food handler	4 (5.1%)	6 (6.7%)	Ref.		Ref.	
Healthcare worker	0 (0%)	2 (2.2%)	-		-	
Nursery/school/high school worker	3 (3.8%)	5 (5.6%)	0.90 (0.13–6.08)	0.91	1.02 (0.14–7.56)	0.99
School/Student	70 (88.6%)	77 (85.6%)	1.36 (0.37–5.03)	0.64	1.34 (0.34–5.24)	0.68
Other *	2 (2.5%)	0 (0%)	-		-	
Hepatitis A vaccinated						
Yes	0 (0%)	1 (0.9%)	-		-	
No	84 (100%)	132 (99.2%)	Ref.		Ref.	
Men who have sex with men ^†^						
Yes	5 (23.8%)	27 (67.5%)	0.15 (0.04–0.50)	0.002	0.23 (0.07–0.82)	0.02
No	16 (76.2%)	13 (32.5%)	Ref.		Ref.	
Drug user ^‡^						
Yes	1 (3.0%)	0 (0%)	-		-	
No	32 (97.0%)	12 (100%)	Ref.		Ref.	

* Performing arts direction, not working, ^†^ Only for men aged ≥15 years, ^‡^ Only for persons aged ≥15 years.

**Table 5:** Characteristics of the first case in common-source and person-to-person transmission hepatitis A outbreaks in regions with different vaccination strategies. Spain, 2010–2018.

	Regions with Universal Vaccination Strategy *n* = 120	Regions with Risk-Group Vaccination Strategy *n* = 330	Crude Odds Ratio (95% CI)	*p-*Value	Adjusted Odds Ratio (95% CI)	*p-*Value
COMMON SOURCE						
Age						
0–14 years	7 (53.8%)	10 (58.8%)	Ref.		Ref.	
15–44 years	4 (30.8%)	6 (35.3%)	0.95 (0.19–4.68)	0.95	0.96 (0.19–4.80)	0.96
≥45 years	2 (15.4%)	1 (5.9%)	2.86 (0.21–37.99)	0.43	2.86 (0.21–38.04)	0.43
Sex						
Male	9 (69.2%)	12 (70.6%)	Ref.		Ref.	
Female	4 (30.8%)	5 (29.4%)	1.07 (0.22–5.14)	0.94	0.98 (0.20–4.94)	0.98
Born in Spain	7 (87.5%)	8 (53.3%)	6.12 (0.60–62.82)	0.13	6.27 (0.47–82.57)	0.16
Occupation						
Food handler	1 (11.1%)	0 (0%)	Ref.		Ref.	
School/Student	7 (11.1%)	3 (100%)	-		-	
Others *	1 (77.8%)	0 (0%)	-		-	
Hepatitis A vaccinated	0 (0%)	0 (0%)	-		-	
Men who have sex with men^†^	2 (100%)	0 (0%)	-		-	
Drug user ^‡^	0 (0%)	0 (0%)	-		-	
PERSON-TO-PERSON TRANSMISSION OUTBREAKS						
Age						
0–14 years	52 (56.5%)	74 (46.5%)	Ref.		Ref.	
15–44 years	31 (33.7%)	69 (43.4%)	0.64 (0.37–1.11)	0.11	0.57 (0.32–1.01)	0.05
≥45 years	9 (9.8%)	16 (10.1%)	0.80 (0.33–1.95)	0.62	0.73 (0.29–1.82)	0.50
Sex						
Male	61 (67.0%)	124 (72.5%)	Ref.		Ref.	
Female	30 (33.0%)	47 (27.5%)	1.30 (0.75–2.25)	0.35	1.18 (0.65–2.13)	0.59
Born in Spain	26 (63.4%)	43 (71.7%)	0.68 (0.29–1.60)	0.38	0.75 (0.30–1.90)	0.54
Occupation						
Food handler	2 (3.4%)	6 (7.1%)	Ref.		Ref.	
Healthcare worker	0 (0%)	2 (2.4%)	-		-	
Nursery/school/high school worker	3 (5.1%)	5 (5.9%)	1.80 (0.21–15.41)	0.59	2.08 (0.22–19.59)	0.52
School/Student	53 (89.8%)	72 (84.7%)	2.21 (0.43–11.37)	0.34	2.27 (0.42–12.31)	0.34
Other ^§^	1 (1.7%)	0 (0%)	-		-	
Hepatitis A vaccinated	0 (0%)	1 (0.9%)	-		-	
Men who have sex with men^†^	3 (16.7%)	27 (71.1%)	0.08 (0.02–0.34)	0.001	0.13 (0.03–0.55)	0.006
Drug user ^‡^	1 (3.7%)	0 (0%)	-		-	

* Not working. ^†^ Only for men aged ≥15 years. ^‡^ Only for persons aged ≥15 years. ^§^ Performing arts direction.

## Data Availability

The data presented in this study are available on request from the corresponding author.

## Supplementary Materials

The following are available online at https://www.mdpi.com/article/10.3390/vaccines9111214/s1 Supplementary Table S1, Number and cumulative incidence rate per million inhabitants of hepatitis A outbreaks in regions with different vaccination strategies. Spain, 2010–2016; and Supplementary Table S2, Characteristics of common source and person-to-person hepatitis A outbreaks in regions with different vaccination strategies. Spain, 2010–2016.

## Appendix A

The PREVICET Working Group of Viral Hepatitis is composed of: Domínguez A, Soldevila N (Universitat de Barcelona and CIBERESP), Varela C, Guerrero M, Peñuelas M (Centro Nacional de Epidemiología and CIBERESP), Torner N (CIBERESP), Martínez A, Godoy P, Borrás E (Agència de Salut Pública de Catalunya and CIBERESP), Rius C (Agència de Salut Pública de Barcelona and CIBERESP), Avellón A (Centro Nacional de Microbiología and CIBERESP), Rodero I (Consejería de Sanidad, Comunidad de Madrid), Carbajal S (Hospital Clínico Universitario de Valladolid), García-Fulgueira A (Departamento de Epidemiología, Consejería de Salud de la Región de Murcia and CIBERESP), Martínez-Pino I (Servicio de Epidemiología, Dirección General de Salud Pública de Castilla y León and CIBERESP), Carbó Malonda RM (Conselleria de Sanitat Universal i Salut Pública de la Comunitat Valenciana), and Rivas AI (Consejería de Sanidad y Consumo de Ceuta).
