# Supplementary material for: Hepatitis A Outbreak Characteristics: A Comparison of Regions with Different Vaccination Strategies, Spain 2010–2018
Source: Vaccines (Basel). 2021 Oct 20;9(11):1214. doi: 10.3390/vaccines9111214 (PMC8620672; doi:10.3390/vaccines9111214)
Supplement: Supplementary file 1 [file vaccines-09-01214-s001.zip › vaccines-1402244-supplementary.pdf]

Supplementary Table S1. Number and cumulative incidence rate per million inhabitants of hepatitis A outbreaks in regions with different vaccination strategies. Spain, 2010-2016

	**Regions with universal vaccination strategy N=93 N (CIR)**	**Regions with risk group vaccination strategy N=180 N (CIR)**	**Rate ratio (95% CI)**	***P* value**
**Outbreak incidence**	12.43	11.32	1.10 (0.85-1.41)	0.46
**Transmission mode**				
Common source	9 (1.20)	13 (0.82)	1.47 (0.60-3.46)	0.38
Person-to-person	71 (9.49)	153 (9.62)	0.98 (0.74-1.30)	0.93
**Setting**				
Family/household	68 (9.09)	142 (8.9)	1.02 (0.76-1.36)	0.90
School	6 (0.80)	17 (1.07)	0.75 (0.27-1.85)	0.56
Leisure facility/summer camp	1 (0.13)	1 (0.06)	2.12 (0.05-82.85)	0.99
Community/other/various	5 (0.67)	16 (1.01)	0.66 (0.22-1.75)	0.44
Other closed facilities	0 (0)	2 (0.12)	0.42 (0.02-8.85)	0.57
**Imported outbreak**	49 (6.55)	47 (2.96)	2.21 (1.48-3.31)	0.001

CIR: cumulative incidence rate

Supplementary Table S2. Characteristics of common source and person-to-person hepatitis A outbreaks in regions with different vaccination strategies. Spain, 2010-2016

	Regions with universal vaccination strategy N=93	Regions with risk group vaccination strategy N=180	Crude odds ratio (95% CI)	*P* value	Adjusted odds ratio (95% CI)	*P* value
**COMMON SOURCE OUTBREAKS**						
**Setting**						
Family/household	8 (100%)	11 (100%)	-		-	
**Imported outbreak**	6 (66.7%)	6 (50.0%)	2.00 (0.33-11.97)	0.45	1.32 (0.10-7.10)	0.47
**No. of exposed people**, median (range)	6 (3-10)	5 (2-56)		0.88		
**No. of affected people**, median (range)	2 (2-5)	2 (2-4)		0.29		
**No. of hospitalized cases**, median (range)	0 (0-2)	2 (0-3)		0.07		
**PERSON-TO-PERSON TRANSMISSION OUTBREAKS**						
**Setting**						
Family/household	51 (83.6%)	119 (77.8%)	Ref.		Ref.	
School	5 (8.2%)	16 (10.5%)	0.73 (0.25-2.10)	0.56	1.67 (0.58-6.66)	0.28
Leisure facility/summer camp	1 (1.6%)	1 (0.7%)	2.33 (0.14-38.03)	0.55	2.50 (0.13-47.52)	0.54
Community/other/various	4 (6.6%)	15 (9.8%)	0.62 (0.20-1.97)	0.42	0.46 (0.10-2.20)	0.33
Other closed facilities	0 (0%)	2 (1.3%)	-		-	
**Imported outbreak**	34 (60.7%)	38 (32.2%)	3.25 (1.68-6.30)	<0.001	3.42 (1.68-6.94)	0.001
**No. of exposed people**, median (range)	6 (2-422)	5 (2-473)		0.48		
**No. of affected people**, median (range)	2 (2-218)	2 (2-24)		0.53		
**No. of hospitalized people**, median (range)	0 (0-2)	1 (0-14)		0.001		
